# Differential uPAR recruitment in caveolar-lipid rafts by GM1 and GM3 gangliosides regulates endothelial progenitor cells angiogenesis

**DOI:** 10.1111/jcmm.12410

**Published:** 2014-10-14

**Authors:** Francesca Margheri, Laura Papucci, Nicola Schiavone, Riccardo D'Agostino, Silvana Trigari, Simona Serratì, Anna Laurenzana, Alessio Biagioni, Cristina Luciani, Anastasia Chillà, Elena Andreucci, Tommaso Del Rosso, Giancarlo Margheri, Mario Del Rosso, Gabriella Fibbi

**Affiliations:** a Department of Experimental and Clinical Biomedical Sciences, Section of Experimental Pathology and Oncology, University of FlorenceFlorence, Italy; b Institute of Complex Systems (ISC), Consiglio Nazionale delle Ricerche (CNR)Florence, Italy; c Department of Physics, Pontificia Universidade Catolica do Rio de JaneiroRio de Janeiro, Brazil; d Istituto Toscano TumoriFlorence, Italy

**Keywords:** angiogenesis, uPAR, GM1, GM3, lipid rafts, caveolar-lipid rafts, endothelial progenitor cells, endothelial colony-forming cells, MAPKinases

## Abstract

Gangliosides and the urokinase plasminogen activator receptor (uPAR) tipically partition in specialized membrane microdomains called lipid-rafts. uPAR becomes functionally important in fostering angiogenesis in endothelial progenitor cells (EPCs) upon recruitment in caveolar-lipid rafts. Moreover, cell membrane enrichment with exogenous GM1 ganglioside is pro-angiogenic and opposite to the activity of GM3 ganglioside. On these basis, we first checked the interaction of uPAR with membrane models enriched with GM1 or GM3, relying on the adoption of solid-supported mobile bilayer lipid membranes with raft-like composition formed onto solid hydrophilic surfaces, and evaluated by surface plasmon resonance (SPR) the extent of uPAR recruitment. We estimated the apparent dissociation constants of uPAR-GM1/GM3 complexes. These preliminary observations, indicating that uPAR binds preferentially to GM1-enriched biomimetic membranes, were validated by identifying a pro-angiogenic activity of GM1-enriched EPCs, based on GM1-dependent uPAR recruitment in caveolar rafts. We have observed that addition of GM1 to EPCs culture medium promotes matrigel invasion and capillary morphogenesis, as opposed to the anti-angiogenesis activity of GM3. Moreover, GM1 also stimulates MAPKinases signalling pathways, typically associated with an angiogenesis program. Caveolar-raft isolation and Western blotting of uPAR showed that GM1 promotes caveolar-raft partitioning of uPAR, as opposed to control and GM3-challenged EPCs. By confocal microscopy, we have shown that in EPCs uPAR is present on the surface in at least three compartments, respectively, associated to GM1, GM3 and caveolar rafts. Following GM1 exogenous addition, the GM3 compartment is depleted of uPAR which is recruited within caveolar rafts thereby triggering angiogenesis.

## Introduction

Gangliosides are neuraminic acid-containing glycosphingolipids and are characteristic components of the plasma membrane of eukaryotic cells, where they typically partition within specialized microdomains called lipid-rafts (LRs), composed by tightly packed sphingomyelin (SM) and cholesterol (chol), as opposed to the other parts of the membrane that are mainly constituted by phospholipids. Such membrane microdomains serve as organizers for the assembly of signalling molecules [1]. Gangliosides are shed from the cell membrane and accumulate in the microenvironment and in plasma, maintaining their property to be efficiently incorporated into the cell membrane [2]. Since tumours shed gangliosides into the microenvironment in greater quantities than do healthy tissues, the potential importance of gangliosides in tumour cell growth and tumour angiogenesis has been thoroughly investigated [3]. Experimental evidence indicates that gangliosides are not angiogenic by themselves, but act synergistically with the main angiogenesis inducers [4,5], even if contradictory data have been reported for both GM1 and GM3 [6,7]. All the experimental approaches included exogenous gangliosides enrichment, but the diversified conditions under which the gangliosides are added to cell cultures may cause different incorporations into the cell membrane, making comparison of results difficult. Therefore, the results from these studies could only be considered as indirect evidence that gangliosides modulate tumour angiogenesis by modulating growth factor signalling. The presence of the urokinase-type plasminogen activator receptor (uPAR) in LRs has been previously reported in human embryonic kidney (HEK)-293 cells [8]. We have shown that endothelial colony-forming cells (ECFCs), a subset of endothelial progenitor cells (EPCs), require uPAR in caveolar-LRs to perform an efficient angiogenic program [9]. Given the reported observation that uPAR is recruited in LRs in HUVEC and its colocalization with GM1 [10], here we have studied the interaction of GM1 and GM3 with uPAR to investigate the possibility of a ganglioside-dependent uPAR recruitment within caveolar-LRs in ECFCs, as well as its functional import in terms of angiogenesis. For these purposes, we first checked the interaction of uPAR with membrane models enriched with GM1 or GM3. In particular, we relied on the adoption of solid-supported mobile bilayer lipid membranes with LR-like composition (solid-supported raft-like membranes, ssRLM) formed onto solid hydrophilic surfaces and evaluated with non-invasive optical tools (surface plasmon resonance, SPR) the amount of uPAR recruited on both enriched ssRLM and, for the first time at our knowledge, we estimated the apparent dissociation constants of uPAR-GM1/GM3 complexes. These observations were validated by identifying a pro-angiogenic activity of GM1-enriched ECFCs, based on GM1-dependent uPAR recruitment in caveolar-LRs. We have found that uPAR is present on the ECFC surface in at least three compartments, one associated to GM1, another associated to GM3 and a third one associated to caveolar-LRs. Following GM1 exogenous addition the GM3 compartment is depleted of uPAR which is recruited within caveolar-LRs thereby triggering angiogenesis-related transduction pathways that eventuate in enhanced ECFC invasion and capillary morphogenesis.

## Materials and methods

### Fabrication of GM1 and GM3-enriched ssRLM and utilization of plasmonic transducers for uPAR adsorption studies

The ssRLMs were assembled by exploiting the lipid vesicles fusion on plasmonic transducers (PTs) that occurs when liposomes are in contact with the hydrophilic interfaces of the 40-nm thick SiO_2_ layers of PTs [11], using the same procedure previously described [12,13]. The resulting ssRLMs had the molar composition GM1_0.1_(GM3_0.1_)SM_0.5_Chol_0.4_ where suffixes 0.1, 0.5, 0.4 refer to 10%, 50%, 40% molar concentration, respectively, in agreement with the known lipid composition of LRs of the eukaryotic cells membranes [14] and of their average GM1/GM3 content [15]. The uPAR (R&D Systems, Minneapolis, MN, USA) solution used for the binding tests had a concentration of 5 μg/ml (8.5 × 10^−8^ M) in HBS. Such a concentration was selected after preliminary experiments aimed at determining the lowest uPAR concentration giving the maximal reflectivity in SPR uPAR-gangliosides association kinetics.

The monitoring of the binding reactions between uPAR and the gangliosides inglobated into the LR-like membranes was performed by using the home made SPR spectrometer which was already described in [13]. The processing of the kinetic data was performed with ORIGIN 8.6 softaware. For the convenience of the reader, we describe the measurement method in Figure S1.

### Endothelial progenitor cells isolation and treatment with gangliosides

Endothelial progenitor cells were isolated from >50 ml human umbilical cord blood of health newborns, essentially as described in [9], upon selection of cord blood units with a number of total nucleated cells <1.3 × 10^9^ (threshold of suitability for the banking established by the Umbilical Cord Bank of Careggi, Firenze, Italy) after maternal informed consent and in compliance with Italian legislation. These cells were referred to as ECFCs previously described [9]. The colonies were mechanically picked from the original plate and seeded onto another gelatin coated-well with EGM-2 (Endothelial Growth Medium, Microtech, Napoli, Italy) 10% FBS for expansion. To enrich the cell membrane with specific gangliosides, semi-confluent (80%) ECFC cultures were washed with HBSS and starved by overnight incubation in EGM-2 2% FBS. ECFCs were washed again and treated for 2.5 hrs with EGM-2 2% FBS containing 5.0 μM GM1 or GM3. Such a concentration was selected on the basis of previous observations [7] and of preliminary data (data not shown) indicating that GMs concentrations between 5 and 10 μM were the most efficient for stimulation (GM1) or inhibition (GM3) of ECFCs invasion. After incubation ECFCs were washed again and utilized for experiments.

### Isolation, characterization and solubilization of ECFC caveolar-LRs

Isolation of caveolar-LR fractions from ECFC lysates was performed with the Caveolae/Rafts isolation kit of Sigma-Aldrich (Milano, Italy), as described [9]. Here, we used ECFC preparations from three different newborns. Lyophilized microdomains proteins were solubilized as previously described [16] and quantified by Bradford method.

### Cell viability assay and *in vitro* parameters of angiogenesis

The viability of ECFCs under various conditions was determined by a cell proliferation assay using the Water-Soluble-Tetrazolium-salt (WST-1) reagent (Roche Italia, Milano, Italy), as previously reported [17]. Invasion was studied in Boyden chambers in which the upper and lower wells were separated by porous polycarbonate filters coated with Matrigel (50 μg/filter) [9]. ECFCs (20 × 10^3^) were placed in the upper compartment of the chamber and migration was evaluated after 6 hrs. Migration was expressed as the absolute number of migrated cells ± SD. *In vitro* capillary morphogenesis was performed in 13-mm tissue culture wells coated with Matrigel, as described [9]. The experimental conditions were the same used for invasion assay. ECFCs were plated (60 × 10^3^/well) in complete EGM-2 medium, supplemented with 2% FCS and incubated at 37°C, 5% CO_2_. Plates were photographed at 6 hrs and at 24 hrs. Six to nine photographic fields from three plates were scanned for each point. Results were quantified taking as 100% the number of alveolar-like structures of the control ± SD.

### Immunofluorescence confocal microscopy

Control and treated ECFCs were grown on coverslips in EGM-2, fixed and permeabilized according to routine immunocytochemistry methods [18]. The anti-human primary antibodies used were: anti-uPAR R3 (1:40, rabbit polyclonal, Santa Cruz, CA, USA), anti-caveolin-1 (1:400; Sigma-Aldrich), anti-GM3 (1:100, mouse monoclonal Ab, Cosmo Bio, DBA Italia, Milano, Italy) Cholera toxin-beta subunit (CTB; 10 μg/ml; Sigma-Aldrich) was used to study GM1 distribution. The secondary antibodies used for single and double immunostainings were: CY3-conjugated antimouse IgG (1:800; C2181; Sigma-Aldrich) and FITC-conjugated anti-rabbit IgG (1:800; F-4151; Sigma-Aldrich). For nuclear staining, samples were incubated with DAPI (2 μg/ml), for 15 min. The mounting procedure and the confocal analysis were run as previously described [8]. Colocalization was determined by ‘Just Another Colocalisation Plugin’ of ImageJ software, as described [9,19].

### Western blot analysis

Western blots were performed on solubilised caveolar-LR and non-LR fractions or on total cell lysates to study the involved transduction pathways. Sample preparation and processing, as well as the WB conditions and gel detection were as previously described [16]. The primary antibodies were: anti-uPAR (1:500, rabbit polyclonal, Santa Cruz), anti-caveolin-1 (1:1000, rabbit polyclonal, Sigma-Aldrich); anti-phospho-ERK (p42/p44) (200 μg/ml, 1:500; rabbit polyclonal, Santa Cruz); anti-ERK-2 (200 μg/ml, 1:500; rabbit polyclonal, Santa Cruz); anti-phospho-p38 (1:500, rabbit polyclonal, Cell Signaling, Danvers, MA, USA); anti-p38 (1:500, rabbit polyclonal, Biosource, Life Technologies Europe, Monza, Italy); anti AKT (200 μg/ml, 1:500; rabbit polyclonal Santa Cruz); anti pAKT (200 μg/ml, 1:500; rabbit polyclonal, Santa Cruz); anti ß1 integrin (200 μg/ml; 1:500, rabbit polyclonal, Santa Cruz).

### Statistical analysis

Results are expressed as mean ± SD. Comparisons were performed by the Student's test, differences were considered statistically significant at *P* < 0.05.

## Results

### uPAR recruitment on GM1 and GM3-enriched ssRLMs

**Fig. 1:**
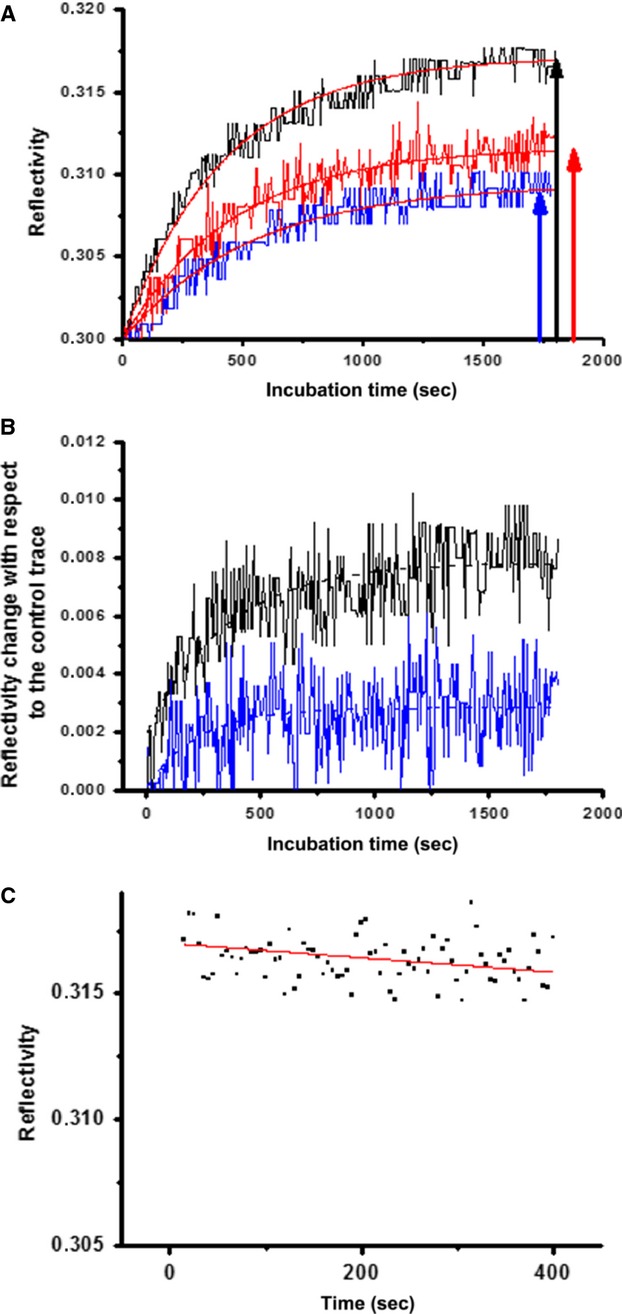
uPAR-gangliosides association/dissociation kinetics evaluated by SPR. (**A**) Association kinetics. Association kinetics of uPAR with ssRLM:GM1 (▬), with ssRLM:GM3 (▬) and with the control GM1,GM3-free ssRLM (▬). (**B**) The association kinetics after the control subtraction. Dashed lines: best fit exponential curves. The values of the association rate constant 4.7 × 10^−3^/sec. and = 1.1 × 10^−3^/sec. for uPAR-GM1 and uPAR-GM3 respectively. (**C**) Dissociation kinetics of uPAR from ssRLM:GM1 as recorded by SPR. Dissociation constant: 9 × 10^−6^/sec. (Error: ±15%).

Fabrication of GM1 and GM3-enriched ssRLMs was first checked by exploiting their affinity with the beta subunit of the Cholera toxin (CTB) [11] and Wheat Germ Agglutinin (WGA) [12], respectively. Once settled the reproducible generation of gangliosides-enriched SM/Chol ssRLM, we examined their affinity for recombinant human uPAR with SPR tests. On the basis of the results obtained in five different uPAR binding experiments, a real affinity between uPAR and ssRLMs:GM1/ssRLMs:GM3 has been revealed, as exemplified by the association sensorgram shown in Figure 1A. Although the reflectivity variation is limited to 0.02, the resolution is good enough to evidence the receptor recruitment. In particular, the amount of uPAR adsorbed on ssRLM:GM1 (dark arrow, Fig. 1A) was twice as much than that on GM1-free ssRLM (the control membranes, blue arrow), while the increase was only 30% for ssRLM:GM3 (red arrow), so that the overall amount of uPAR adsorbed on GM1-rich ssRLM is 2.6 times that in presence of GM3. It is worth noticing that the adsorption of uPAR on the ssRLM occurs even in the absence of its GPI anchor, which is the physiological linker of uPAR to the cell membrane.

The association rate constants *k*_*on*_ were calculated from a monophasic exponential best fit of the traces reported in Figure 1B obtained after the subtraction of the control kinetics from those of Figure 1A. Because of the small response of the sensor, the difference traces are very noisy, and allow only a rough estimate of *k*_*on*_ values, that were calculated to be (4.7 ± 0.9) × 10^−3^/sec. and (1.1 ± 0.2) × 10^−3^/sec. for uPAR-GM1 and uPAR-GM3 respectively. The association constants *k*_*ass*_ = (5.5 ± 1.0) × 10^4^/M/sec. and *k*_*ass*_ = (1.3 ± 0.3) × 10^4^/M/sec. are readily found. After two rinsing cycles, in the presence of the sole HBSS, we tried to measure the dissociation constants of the complexes, starting from GM1-uPAR. Rather than the expected decline of the reflectivity, we noticed further very slow increases of the signal, likely because of reassembling of uPAR on the ssRLM [20]. Nevertheless, for at least one sample of ssRLM:GM1, the rearrangement occurred in a shorter time, that allowed us to record the subsequent uPAR dissociation, whose track is reported in Figure 1C. Its best fit permits to calculate *k*_*diss*_, that results (9.0 ± 1.9) × 10^−6^/sec. Considering the corresponding association constant, the value of *K*_*D*_ = (1.6 ± 0.6) × 10^−10^ M is found for the apparent dissociation constant [12]. Even if a similar evaluation for ssRLM:GM3 could not be pursued, nevertheless the steady behaviour of the SPR traces (not reported) evidence that uPAR-GM3 complexes are quite stable. Aiming to obtain also the ssRLM:GM3-uPAR dissociation constant and a more precise *k*_*diss*_ for the ssRLM:GM1-uPAR system, we switched to a different kind of optical transducers, namely a plasmon waveguide resonator (PWR), widely discussed for the good of the interested reader in the Supplementary Material. The *K*_*D*_ value found for uPAR-GM1 complex (5.3 ± 0.4) × 10^−10^ M/sec., is approximately fourfold that one (1.30 ± 0.26) × 10^−10^ M, found for uPAR-GM3.

### Exogenous addition of GM1 and GM3 modifies the phenotypic behaviour of ECFCs

**Fig. 2:**
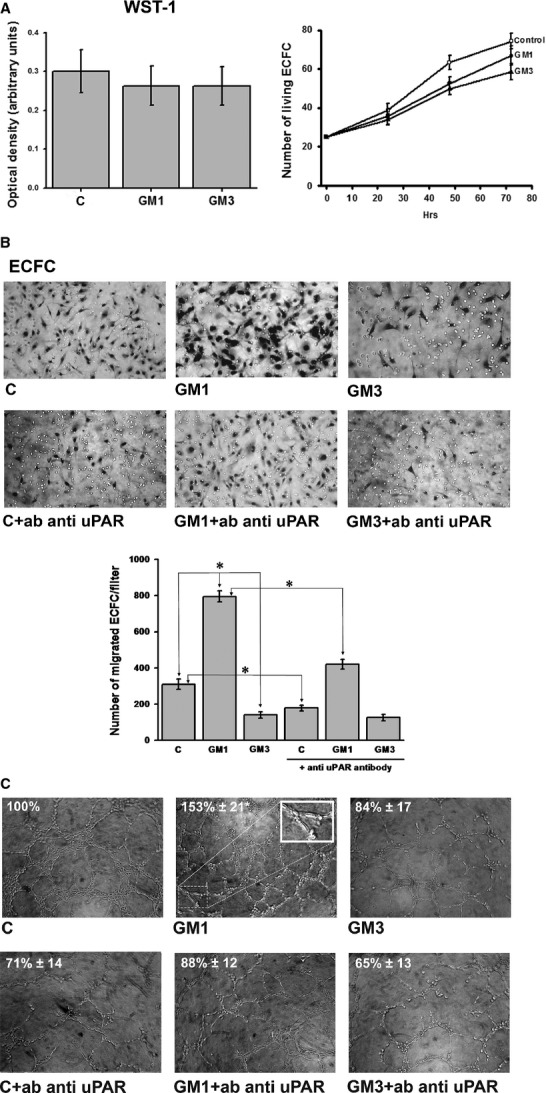
Phenotypic effects of ganglioside enrichment of ECFCs. (**A**) WST-1 cell viability assay (left) and proliferation of viable cells (right) (C = control). (**B**) Matrigel invasion under control conditions and in the presence of gangliosides ± anti- uPAR antibody. (**C**) Capillary morphogenesis under control conditions and in the presence of gangliosides ± anti- uPAR antibody. Capillary tube formation (shown at higher magnification in the inset) was quantified, taking as 100% the number of alveolar-like structures of the control (values are shown within pictures ± SD). Magnification 100X. Each point of each experiment was performed in triplicate with three different ECFC lines and values are expressed ±SD. *P < 0.05, significantly different from control ECFCs.

Given the observed high affinity of gangliosides-uPAR interaction and the known importance of uPAR and gangliosides in angiogenesis, we studied the pro-angiogenic effects of exogenous gangliosides addition to ECFCs, as related to uPAR function and distribution. Angiogenesis *in vitro* is usually studied by evaluating endothelial cell invasion of a reconstituted physiological matrix (Matrigel invasion), which is representative of the very first step of endothelial cells recruitment within angiogenesis matrices, and the formation of tubular-like structures within the same matrix (capillary morphogenesis). Upon verification that GM1/3 treatment did not affect ECFC viability and proliferation, as shown by WST-1 assay and counting of viable cells (Fig. 2A), we studied their angiogenesis properties. While GM3 treatment produced a decrease in ECFC Matrigel invasion, GM1 enrichment resulted into an increase in the same parameter (Fig. 2B). Similar results were obtained in capillary morphogenesis induction experiments, where GM1 addition produced an increase in capillary-like tubules, while GM3 induced a decrease with respect to the spontaneous activity of control untreated ECFCs (Fig. 2C). The function of uPAR in GM1/3-dependent capillary morphogenesis was evaluated in the presence of an uPAR-blocking antibody, that produced inhibition of tubular-like structures formation in both control and GM1-treated ECFCs, while it did not affect the already reduced morphogenetic properties of GM3-treated ECFCs (Fig. 2C). These data, while indicating the relevance of uPAR in GM1-dependent angiogenesis, also suggest that the negative effect of GM3 could possibly depend on the scarce availability of an uPAR pool functionally suitable to perform a proper angiogenesis program.

### GM1 and GM3 enrichment of ECFCs differentially regulates uPAR recruitment in caveolar-LRs and uPAR-dependent signalling pathways in ECFCs

**Fig. 3:**
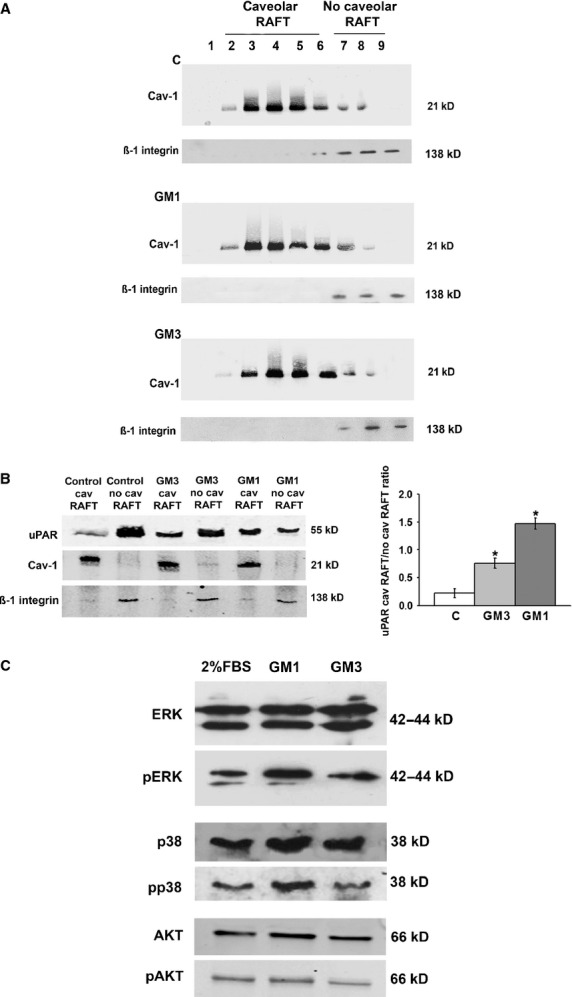
Ganglioside treatment of ECFCs: uPAR distribution in lipid-raft and non-raft fractions and MAPKinases phosphorylation. (**A**) Western blot of caveolin-positive (caveolar-LRs) and β1-integrin-positive fractions (no-caveolar-LRs) obtained by density gradient centrifugation under control conditions and ganglioside treatment. (**B**) Western blot with uPAR antibody of collected caveolar-LR fractions (identified by the positive blotting for caveolin-1) and no-caveolar-LR fractions (identified by the positive blotting for β1-integrin). The blot shown is representative of three different experiments performed in three different ECFC preparations. The right side of panel B show the densitometry quantification of uPAR, normalized for the caveolar-LR and no-caveolar-LR markers. (**C**) Western blot of total and phosphorylated forms of the MAPKinases ERK, p38 and AKT, representative of three different experiments performed in three different ECFC preparations in confluent ECFC in the presence of 2% FCS (the standard culture conditions of ECFCs) and following 20 min. of 5 μM gangliosides addition. In each blot MW are reported on the left.

Extracts of ECFCs under control conditions and after gangliosides treatment were subjected to density gradient separation, as described [9,16]. Fractions were assayed by Western blotting for the presence of caveolin-1 as caveolar-LR marker (fractions 2–6) and of ß1-integrin as non-caveolar-LR marker (fractions 7–9). The distribution of caveolin-rich fractions did not differ between control and gangliosides-treated ECFCs (Fig. 3A). Therefore, we collected separately the caveolar-LR and non-caveolar-LR fractions from three different experiments with three different ECFC lines and performed an electrophoretic separation of caveolar-LR and non-caveolar-LR proteins followed by blotting with an anti-uPAR antibody. In control ECFCs uPAR was detectable almost exclusively in the non-caveolar-LR fraction, while it shifted to the caveolar-LR fraction following treatment with both GM1 and GM3 (Fig. 3B). However, as evident in the shown blotting and in the histogram, the caveolar-LR uPAR enrichment resulted much higher following GM1 treatment than upon GM3 addition.

Several uPAR signalling pathways are activated in different cell types, leading to the description of an ‘uPAR signalosome’ which may differ among various cells, depending on the uPAR partner molecule capable of signalling [21]. However, available evidence indicate that uPAR-dependent angiogenesis pathways mainly involves MAP-kinase activation to produce p-ERK and p-p38 [9,22]. Our Western blotting analysis, performed on ECFCs lysates after 20 min. of GM1/GM3 addition, revealed that p-ERK and p-p38 were up-regulated only by GM1, while p-AKT production (another typical phosphorylated molecule of the uPAR signalosome) did not show any appreciable variation (Fig. 3C). These data are in agreement with the GM1-enhanced localization of uPAR with caveolar-LRs that are the most important signalling platforms in uPAR angiogenesis in ECFCs [9,16].

### Confocal analysis of uPAR, GM1, GM3 and caveolin after GM1 and GM3 enrichment of ECFCs

To support physical, functional and biochemical observations with structural features, we performed confocal analysis of GM1, GM3, uPAR and caveolin.

**Fig. 4:**
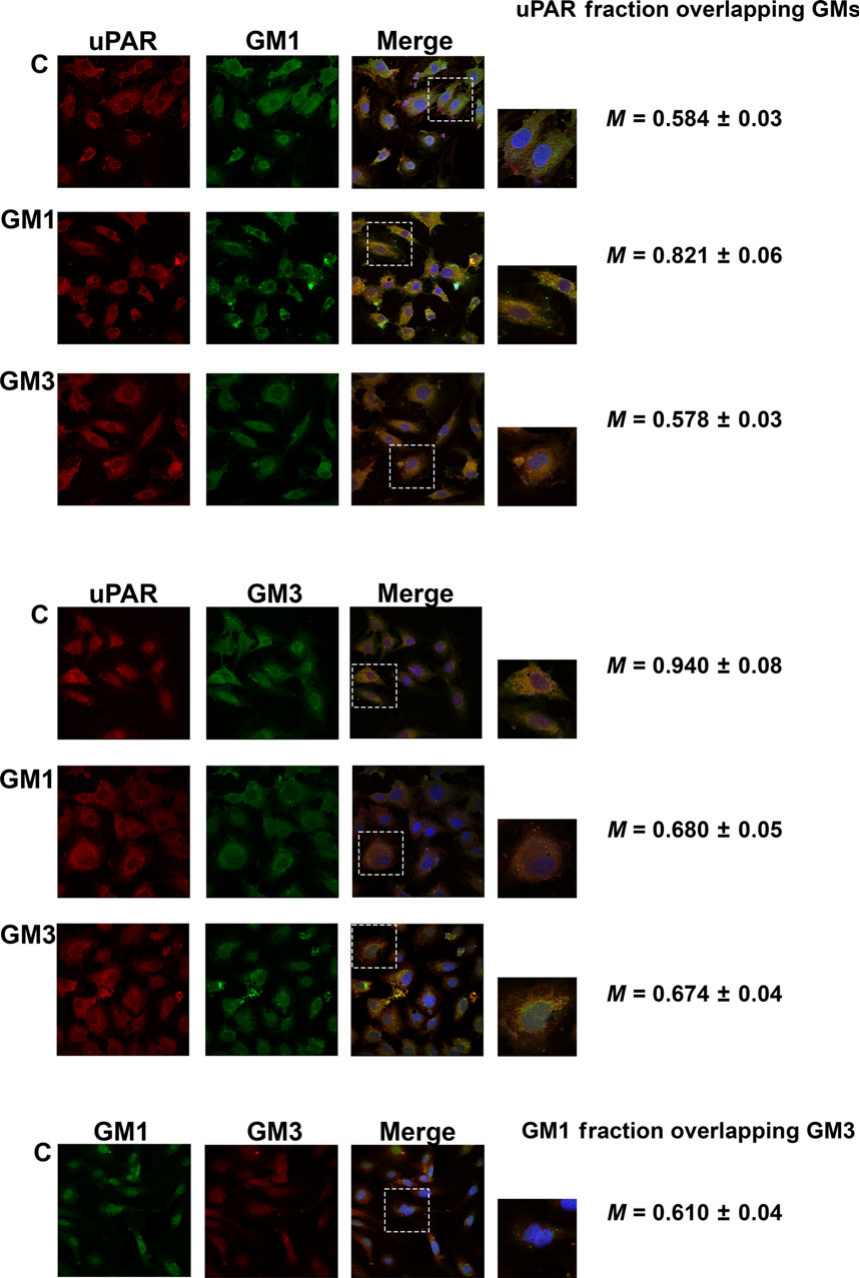
Confocal study of GM1, GM3 and uPAR in ECFCs. Colocalizations of uPAR (Red stain) with GM1 (Green stain) or uPAR with GM3 (Green stain) are in upper and lower panel respectively. Treatments with gangliosides are indicated on the left side (C = Untreated Control) of the images. On the right sides of the images, the Mander's coefficient is reported for each treatment. Both GM1 and GM3, clearly induce uPAR and GM1 colocalization, displacing uPAR from the GM3 compartment. In basal conditions, GM1 and GM3 show minimal colocalization (M = Mander's coefficient). The images shown were selected out of 60 images for each condition obtained in two different ECFC lines in three different experiments (magnification 60X). The magnification of insets is 140X.

On the basis of our SPR data indicating a 10^−10^ M K_D_ for GM1/3-uPAR interaction, we first studied GM1, GM3 and uPAR localization in ECFCs by confocal microscopy. Under basal conditions both GM1 and GM3 colocalized with uPAR, but GM3 showed an almost total colocalization (M = 0.940), while GM1 exhibited only a minimal uPAR colocalization (M = 0.584; Fig. 4). This data, coupled with the results shown in Figure 3B, indicates a preferential GM3-dependent uPAR trapping in non-caveolar-LR fractions of the cell membrane under control conditions. Treatment with GM1 and GM3 clearly induced uPAR colocalization with GM1 (M = 0.821), displacing uPAR from the GM3 compartment (M = 0.680 and 0.674 respectively; Fig. 4), thereby confirming the higher uPAR-GM1 affinity observed in SPR experiments. Under basal conditions GM1 and GM3 showed a scarce colocalization (M = 0.610).

**Fig. 5:**
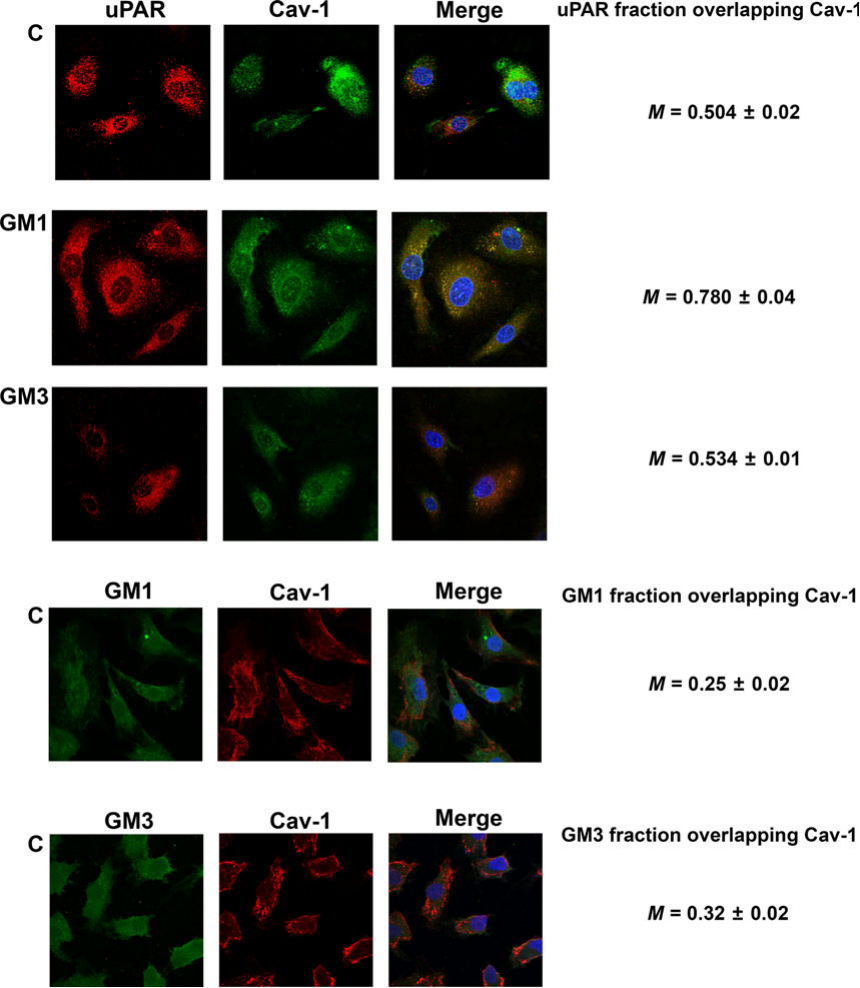
Confocal study of Caveolin and uPAR in ECFCs. The pictures show colocalization of uPAR (Red stain) with Caveolin-1 (Green stain). Treatments with gangliosides are indicated on the left side (C = Untreated Control) of the images. On the right sides of the images, the cytofluorogram is reported for each treatment. GM1, but not GM3, clearly recruits uPAR to caveolae (magnification 60X).

Since the analysis of uPAR distribution in caveolar-LR and non-caveolar-LR fractions following GM1 and GM3 ECFC enrichment showed a preferential caveolar-LR uPAR homing after GM1 stimulation, we also studied caveolin-1-uPAR colocalization by confocal microscopy upon GM1 and GM3 ECFC enrichment. These experiments clearly indicated that GM1 but not GM3 recruits uPAR to caveolae, as indicated by merged images and by Mander's coefficients reported on the right of each lane (Fig. 5). The Image J analysis shown in Table 1 not only indicated a strong GM1-dependent increase in uPAR-caveolin colocalization with respect to both control and GM3-treated ECFCs but also showed that GM1 treatment induced higher numbers and larger areas of caveolar-LR clusters.

**Table 1:** Confocal analysis of uPAR and caveolin 1

Confocal analysis EPC	Maximum caveolar raft, cluster area, μm^2^ (based on Cav-1 signal)	Caveolar raft, cluster number (based on Cav-1 signal)	Colocalization (uPAR fraction overlapping Cav-1)
CTRL	3.259 ± 0.12	17.00 ± 0.10	0.504 ± 0.02*
GM1	7.00 ± 0.49 (*P* < 0.05†)	144.9 ± 11.91 (*P* < 0.05†)	0.780 ± 0.04* (*P* < 0.05†)
GM3	4.98 ± 0.34 (*P* < 0.05†)	78.9 ± 6.23 (*P* < 0.05†)	0.534 ± 0.01*

* Mander's coefficients (0 = absence of colocalization; 1 = total colocalization).

† *P* indicates significance of differences between control and the relevant parameters.

Analysis performed by Image J software of image in Figure 5: caveolar-raft clustering, number of clusters and uPAR-CAV-1 colocalization are reported. GM1 treatment induces caveolar-cluster formation as deduced by increased cluster area and cluster number. GM3 has a lower power to induce caveolar clustering and does not induce Cav-1 and uPAR colocalization.

It is noteworthy that GM1 and GM3 do not colocalize with each other or with caveolin (Figs 4 and 5), as already reported in breast cancer cells and in canine kidney epithelial cells [23,24].

Overall, these data indicate that membrane uPAR is distributed in at least three compartments in ECFCs: the first one associated to caveolar-LRs, the second to GM1 and the third to GM3. This is deducible by the evidence that (*i*) caveolin does not colocalize with GM1 and GM3, (*ii*) GM1 and GM3 show minimal colocalization, while (*iii*) uPAR colocalizes with all of them. Most importantly, only GM1 treatment stimulates a relevant recruitment of uPAR in caveolar LRs, MAPKinases signalling, invasion and capillary morphogenesis of ECFCs.

## Discussion

In this study, we have shown by SPR that GM1 and GM3 embedded in artificial ssRLM efficiently bind soluble uPAR, exhibiting very high affinity (*K*_*D*_ ∼10^−10^ M). Moreover, SPR and PWR measurements have shown that GM1 binds to uPAR more effectively than GM3, so that GM3-uPAR complexes are likely to detach in presence of a proper enrichment of competing GM1. On these basis, we planned to reconcile our data with the data of literature showing that GM1 promotes angiogenesis [4–6] while GM3 does not, exhibiting an anti-angiogenesis activity under some experimental settings [5], and that uPAR localization in caveolar-LRs is indispensable for an efficient angiogenesis program in EPCs/ECFCs [9,16], commonly considered the main source of vasculogenesis in human tumours [25]. We have shown that uPAR present on ECFC surface is distributed in at least three compartments, respectively associated to caveolae, GM1 and GM3. The three compartments are not static rather they can modify their uPAR content to satisfy angiogenesis needs. In fact, we have shown that under resting control conditions uPAR is mainly localized in GM3 non-caveolar-LR compartment of the cell membrane (Figs 3B, 4 and 5); GM1 enrichment increases the uPAR-GM1 compartment by producing a detachment of uPAR from the G3 compartment (Fig. 4), a result that is in agreement with the higher affinity of GM1 measured with the optical tests on ssRLMs; GM1 treatment stimulates uPAR localization in caveolar-LRs (Figs 3B and 5), promotes ECFC invasion and capillary morphogenesis that are inhibited by uPAR-blocking antibodies, and stimulates the pro-angiogenesis-related ERK and p38 phosphorylation. These data account for the already reported yin-yang activities of gangliosides in angiogenesis, with GM1-dependent angiogenesis counterbalanced by GM3 anti-angiogenesis [4–6], and define a so far unknown property of GM1 to promote a caveolar-LR enrichment of uPAR. We have previously reported that uPAR localization in caveolae is indispensable for ECFC angiogenesis both *in vitro* and *in vivo* [9]. As shown also in the present study, some authors demonstrated that uPAR can be found both in caveolar-LR and non-caveolar-LR fractions and that uPAR distribution in different fractions was associated with different signalling pathways and, consequently, biological processes [8,26,27]. Caveolae are functionally and morphologically distinct forms of LRs characterized by the presence of the protein caveolin-1 and are particularly abundant in endothelial cells, playing a fundamental role in their function [28]. VEGF localizes in caveolae upon interaction with its type-2 receptor (VEGFR2), forming a ‘functional platform’, together with other signalling molecules, such as uPAR, involved in angiogenesis [16,29].

On the basis of these data and of our observations, we suggest that GM1-dependent localization of uPAR in caveolar-LRs accounts for GM1-dependent angiogenesis.

GM1 activity promoting caveolar-LRs localization of uPAR also accounts for the reported synergy between GM1 and classical angiogenesis inducers, such as basic FGF2 and VEGF [4,7], since both promote uPAR recruitment in caveolar-LRs [this study and nine]. Several data show that uPAR represents a central mediator of growth factor-induced endothelial cell migration and undergoes up-regulation and caveolar-LR localization following pro-angiogenesis factors challenge of endothelial cells and ECFCs [9,22,30]. Therefore, it is likely that GM1 and angiogenesis factors cooperate in caveolar-LRs uPAR partitioning, thus lowering the amount of pro-angiogenetic molecules required to trigger an efficient angiogenesis program.

In tumour cell lines, the tumourigenic potential correlates with the cellular levels of gangliosides [31] and the ability to form experimental tumours can be affected by the artificial manipulation of tumour ganglioside levels [32], indicating tumour GM3 overexpression as a determinant of non-aggressive tumour behaviour. The anti-angiogenesis properties of GM3 and its inability to stimulate a caveolar-LR localization of uPAR strongly support these observations. Moreover, many tumours release high amounts of complex gangliosides, such as GM1 [33]. In this types of tumours GM1 may exert its pro-angiogenic activity either alone, as shown by our data, or in synergism with pro-angiogenesis factors. An interesting study has recently shown that GM1 gangliosides recruit tumour-produced soluble uPAR (suPAR) on HUVEC LRs and that such a recruitment triggers an angiogenesis program, thus enlightening a so far unknown mechanism of tumour angiogenesis [10].

Although many studies show that cholera toxin, and therefore GM1, is associated with caveolae and LR domains in various cell types and that cholera-toxin can be internalized by caveolar-LR-dependent endocytosis, we did not find any colocalization between caveolar-LRs and GM1 in ECFCs. The lack of association between GM1 and caveolar-LRs has been reported previously in other cell types, such as canine kidney epithelial cells and mammary epithelial tumour cells [23,24], but has never been described before in endothelial cells or in EPCs. It is evident that these features involve an indirect mechanism of GM1-dependent caveolar-LR distribution of uPAR. A possible explanation for GM1-dependent uPAR localization in caveolar-LR in the absence of a caveolar-LR localization of GM1 in ECFCs may be found in the reported property of non-caveolar-LR GM1 to mediate endocytosis of cholera toxin-B in a dynamin-dependent but caveolar-independent fashion [23]. On this basis, one may hypothesize that, once internalized, uPAR and GM1 follow different pathways within the extremely complex intracellular trafficking network, leading uPAR to caveolar-LR localization, as previously described for constitutive endocytosis and recycling of uPAR [34].

Finally, the occurrence of an agreement between the activities of the gangliosides *versus* uPAR when embedded in biomimetic membranes and on living cells, recently documented for the GM1 affinity with the β-subunit of the Cholera toxin [12], is an important confirmation of the predictive properties of the novel mobile raft-like macromembranes, proposed in [13] and here fruitfully exploited as guideline for more targeted biological investigation on living cells.

## Supporting information

**Data S1** Plasma waveguide resonator (PWR) technology.
