# Supplementary material for: Differential uPAR recruitment in caveolar-lipid rafts by GM1 and GM3 gangliosides regulates endothelial progenitor cells angiogenesis
Source: J Cell Mol Med. 2014 Oct 14;19(1):113–23. doi: 10.1111/jcmm.12410 (PMC4288355; doi:10.1111/jcmm.12410)
Supplement: Supplementary file 1 — Data S1 Plasma waveguide resonator (PWR) technology. [file jcmm0019-0113-sd1.doc]

**Supplemental material**

**1- Optical measurement of association-dissociation kinetics**

The monitoring of the formation of ssRLM, extensively discussed in [1SM] and the uPAR association-dissociation kinetics, were performed using a home-made SPR spectrometer. This instrument measures the light reflected by a plasmonic optical element when its external surface is probed by an evanescent electromagnetic wave that propagates parallel to it. The probe wave is coupled to the transducer via the classical Kretschmann scheme [2SM] depicted in **figure 1SM** where the light enters a coupling glass prism and hits the sensing element, namely a glass slide covered with an Au/SiO_2_ bilayer, at an incidence angle *θ*. Spanning on *θ * above the critical angle relative to prism-solution media, the reflectivity drops down in correspondence to a particular angle, usually referred to as the resonance angle *θ_res_*, giving rise to the so-called phenomenon of Attenuated Total Reflection (ATR). When a certain mass amount accumulates on the sensor surface, the resonance angle *θ_res_* suffers a shift that is directly proportional to the mass, and, if properly calibrated, it can be used to determine the quantity of substance adhering atop the sensing surface. In another measurement route, the incidence angle is kept at a fixed value *θ_0_*  and the reflectivity is monitored in time. In this case, the mass variation determines an increase of reflectivity that is directly proportional to it as far as the response is small in comparison to the sensor linear range [3SM].

In a typical Surface Plasmon Resonance (SPR) transducer (usually referred as plasmonic transducer PT), the optimal coupling is obtained with a 50 nm gold thickness, while, for the quartz layer, we adopted a thickness of 40 nm. In our binding experiments of uPAR to ssBLM:GM1(GM3) performed with SPR transducers, we found that the association of uPAR with ssBLM ssBLM determines a maximum increase of 0.0165 absolute reflectivity (1.5 %) for GM1-uPAR and 0.0105 for GM3-uPAR, indicating the higher tendency of uPAR to bind to GM1-enriched ssRLM. In spite of the high level of noise, the adsorption of uPAR is evidenced, and, though with remarkable errors, values of the association constants *k_ass_* could be obtained for both the uPAR-GM1(GM3) complexes. On the contrary, the dissociation kinetics exhibited too small variations to infer trusty values of the dissociation constants *k_diss_*.

In order to measure *K_D_* for both complexes and to obtain a more accurate comparison between the amounts of uPAR recruited onto GM1(GM3)-ssRLM, we exploited an alternative structure where the SiO_2_ layer’s thickness is increased up to hundreds nanometers.

In this way we obtained a metal-dielectric optical waveguide (mostly known as Coupled Plasmon Waveguide Resonator, or simply Plasmon Waveguide Resonator, PWR) [4SM] with the light power mainly confined in the transparent dielectric. This feature enables the guided electromagnetic wave to propagate for a longer distance than in the PTs, where, on the contrary, the energy is concentrated in the metal layer suffering heat losses and consequent fast damping. This difference in the propagation distances ultimately accounts for the superior resolution of PWRs compared to PTs, as it can be inferred from Eq. 24 of [5SM]. In the PTs case the probed path is typically very small (for example using gold it is 4 μm @ 632.8 nm wavelength), and much higher for PWRs (>60 μm @ 632.8 nm wavelength). A more direct comparison of the sensing performances can be reliably performed by comparing their Figure Of Merit (*FOM*), given by the ratio between the sensitivity, defined as *Δθ_res_* / *Δn_substrate_*, where *Δn_substrate_* is the refractive index variation of the substrate, and the width of the reflectivity curve [6SM]. As schematically depicted in **figure 2SM**, a higher *FOM* corresponds to a higher rate of change *r* = *ΔR/Δs* of the reflectivity change (*ΔR*) vs. the adlayer growth (*Δs*) when the sensor is illuminated at a fixed incidence angle.

The increased resolution of PWR is obtained at expenses of a decrease of the dynamic range and the linearity range of the sensor, lower than that of the SPR transducers. The resolution also increases with the *S/N* of the electronic apparatus that converts the light intensity into a processable electronic output.

We designed a structure of Au/SiO_2_ PWR where Au and SiO_2_ thicknesses are 45 and 500 nm respectively, choosing for the optical dielectric constants among the values found in [1SM ], namely -11.74 + 1.47 i  for gold and 2.10 for SiO_2_.


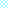


**Incubation cell**

Interrogation beam

**Figure 1SM.** Principle of the measurement. The impinging light beam hits the interrogation area and is coupled to the optical transducer, a gold-quartz bilayer. Spanning on the incidence angle *θ ,* the reflectivity drops to a minimum in correspondence to the resonance angle (*θ_res_* ), that shifts to higher values when mass accumulates on the quartz-solution boundary. At a fixed incidence angle *θ_0_ *, the reflectivity increases in time correspondingly.

Optical transducer

**Reflectivity**

**Gold**

**SiO_2_**

*θ_res_*

*θ_0_*

*θ*

***Incidence angle***

**Time**

**2- Association-dissociation tests of uPAR to/from ssBLM:GM1(GM3)**

We fabricated PWR samples with the same route adopted to fabricate the PTs.

The best fit of the PWR spectra allowed us to calculate the rate *r*, that resulted 2.2-fold lower (13.1 % nm^-1^) than the design value (27% nm^-1^), but still 7.2-fold higher than that calculated for the PT (1.8 % nm^-1^).

These expectations were tested with experiments of association between ssRLM:GM1 and uPAR in PT and PWR. The GM1 had a 10% molar concentration, while uPAR had a 5 μg/mL (8.5x10^-8^ M) concentration in HBS solution. The resulting association kinetics are shown in **figure 3SM**. In the case of PWR, the reflectivity variation after 1700 sec incubation is ~ 8-fold higher than that found using PTs, in nice agreement with the value expected for the fabricated PWR.

Afterwards, we used other PWR samples to perform the uPAR association-dissociation experiments to/from ssRLM:GM1 (GM3) and ganglioside-free ssRLM, namely the control bilayers. As expected, the higher resolution of PWRs allows the evaluation of *k_diss_* also in the case of the detachment of uPAR from ssRLM:GM3.

The values of the association-dissociation constants, as calculated by their best-fitting after the control traces subtraction, are given in the text and in the captions of the figures.

The sensorgrams, exemplified by the tracks reported in **figure 4SM**, confirmed that ssRLMs:GM1 bind a higher mass of uPAR, that resulted 3.6-fold higher than that bound onto ssRLM:GM3, ratio that is of the same order of magnitude of that, 2.6, measured with SPR transducers. This fact suggests that the capturing capability of the fabricated GM1(GM3)- enriched ssRLMs is reasonably reproduced onto our home made SiO_2_ depositions. Moreover, the exponential best fitting of the association kinetics on PWR-supported ssRLMs, led to the values of the association rate constants of  *k_on_*= (8.0±0.3)x10^-3^ s^-1^ and *k_on_*= (2.3±0.3)x10^-3^ s^-1^, corresponding to *k_ass_* = (9.4±0.4)x10^4^ M^-1^s^-1^ and *k_ass_* = (2.7±0.4) x10^4^ M^-1^s^-1^ for ssRLM:GM1 and ssRLM:GM3 respectively, that are of the same order of those found in the SPR tests.

After the rinsing cycles, we recorded the dissociation tracks of uPAR in presence of the sole HBS. Using PWRs, the obtained tracks (**figure 5SM**) were nicely fitted with an exponential decaying kinetic model, that led to the evaluation of the dissociation constants *k_diss_* = (3.5±0.3)x10^-6^ s^-1^ and *k_diss_* = (4.2±0.2)x10^-6^ s^-1^ for the dissociation of uPAR from ssRLM:GM1(GM3) respectively. The values *K_D_* = (5.3±.0.4)x10^-10^ M and *K_D_* = (1.30±0.26)x10^-10^ M are readily achieved for the apparent dissociation constants of ssRLM:GM1(GM3) respectively. It is worth noticing that these values represent upper estimates of the apparent *K_D_*  of gangliosides-uPAR complexes, as we couldn’t separate the contribution to *k_diss_* due to the dissociation of uPAR from the ganglioside-free ssRLMs. Despite this limitation, these values are at our knowledge the first estimations of the binding strength between uPAR and GM1, GM3 hosted in a physiological-resembling lipid matrix .

In the case of ssRLM:GM1, the found values of *K_D_=* (5.3±.0.1)x10^-10^ M obtained with PWR and that, (1.6±.0.6)x10^-10^ M obtained with SPR transducers, indicate that, at least for GM1, a good reproducibility of the binding capability of our ssRLM.

***Reflectivity***

***Reflectivity***

(*a*)

(*b*)

***n***

***Incidence angle***

***n+Δ*** ***n***

***n***

***n+Δ*** ***n***

***Incidence angle***

**Low FOM**

**High FOM**

**Figure 2SM.** Reflectivity vs. incidence angle (angular spectra) for (a) SPR and (b) PWR transducers when the same mass increase occurs at the sensor interface. In both cases, the interface refractive index changes from *n* to *n+Δ* *n* . This variation is accompanied by a right shift of the spectrum (**→**), that is higher in the case of an SPR transducer. However, the width of the angular spectrum is much lower for PWR system, with an overall higher figure of merit (*FOM,* see text for the definition), that accounts for the higher reflectivity variation.

**Figure 3SM.** Kinetics of uPAR adsorption on ssBLM:GM3 grown onto a PT and on a PWR. The initial reflectivity is set to 0.3. The reflectivity variation at the end of the association kinetic on PWR is ~ 8-fold higher than that one recorded with PT.

**Figure 4SM.** Association kinetics of uPAR to ssRLM:GM1 (black line), ssRLM:GM3 (red line) before (a) and after (b) the control (ganglioside-free ssBLM, blue line) subtraction, generated onto a PWR. In (b) the dashed lines are the exponential best fits with association rate constants of *k_on_*= (8.0±0.3)x10^-3^ s^-1^ (ssRLM:GM1) and *k_on_*= (2.3±0.03)x10^-3^ s^-1^(ssRLM:GM3).

a

b

a b

**Figure 5SM.** Dissociation kinetic of uPAR from (a) ssRLM:GM3 and (b) ssRLM:GM1 (black points) and their best fit curve (red lines), corresponding to dissociation constants *k_diss_* = (3.50±0.15)x10^-6^ s^-1^ and *k_diss_* = (4.2±1.5)x10^-6^ s^-1^ respectively.

REFERENCES

1. **Margheri G, D'Agostino R, Del Rosso M, Trigari S.** Fabrication of GM3-enriched Sphyngomyelin/Cholesterol solid-supported lipid membranes on Au/SiO_2_ plasmonic substrates. *Lipids*. 2013;48:739-47.
2. **Raether H** (1981) Surface plasmons on smooth and rough surfaces and on gratings. Springer-Verlag, Berlin.
3. **Horn N, Kreiter M.** Plasmon spectroscopy : methods, pitfalls and how to avoid them. *Plasmonics*. 2010;5:331-345.
4. **Salamon Z, Angus McLeod H, Tollin G.** Coupled plasmon waveguide resonators: a new spectroscopic tool for probing proteolipid film structure and properties. *Biophysical J.* 1997;73:2791-2797.
5. **Lukosz W.** Principles and sensitivities of integrated optical and surface plasmon sensors for direct affinity sensing and immunosensing. *Biosensors & Bioelectronics.* 1991;6:215-225.
6. **Homola J.** Surface Plasmon resonance sensors for detection of chemical and biological species. *Chem Rev.* 2008;108:462-493.
